# Association of KIR Genes with Middle East Respiratory Syndrome Coronavirus Infection in South Koreans

**DOI:** 10.3390/jcm13010258

**Published:** 2024-01-02

**Authors:** In-Cheol Baek, Eun-Jeong Choi, Hyoung-Jae Kim, Haeyoun Choi, Hyoung-Shik Shin, Dong-Gyun Lim, Tai-Gyu Kim

**Affiliations:** 1 Catholic Hematopoietic Stem Cell Bank, College of Medicine, The Catholic University of Korea, Seoul 06591, Republic of Korea; icbaek@catholic.ac.kr (I.-C.B.); cutiejeong@catholic.ac.kr (E.-J.C.); sky1119@catholic.ac.kr (H.-J.K.); chlgosl@catholic.ac.kr (H.C.); 2 Department of Microbiology, College of Medicine, The Catholic University of Korea, Seoul 06591, Republic of Korea; 3 Department of Infectious Diseases, College of Medicine, Eulji University, Daejeon 34824, Republic of Korea; hyoungsshin@gmail.com; 4 Translational Research Center, Research Institute of Public Health, National Medical Center, Seoul 04564, Republic of Korea

**Keywords:** KIR, HLA, polymorphism, MERS, immunogenetics, Koreans

## Abstract

Background: Middle East respiratory syndrome (MERS) is a lower respiratory tract disease caused by a beta coronavirus (CoV) called MERS-CoV, characterized by a high mortality rate. We aimed to evaluate the association between genetic variation in killer cell immunoglobulin-like receptors (KIRs) and the risk of MERS in South Koreans. Methods: KIR genes were genotyped by multiplex polymerase chain reaction with sequence-specific primers (PCR-SSP). A case-control study was performed to identify the odds ratios (OR) of KIR genes for MERS and the association of KIR genes and their ligands, human leukocyte antigens (HLA) genes. Results: KIR2DS4D and KIR3DP1F showed higher frequencies in the group of all patients infected with MERS-CoV than in the control group (*p* = 0.023, OR = 2.4; *p* = 0.039, OR = 2.7). KIR2DL1, KIR2DP1, and KIR3DP1D were significantly associated with moderate/mild (Mo/Mi) cases. KIR2DL2, KIR2DS1, and KIR3DP1F were affected in severe cases. When we investigated the association between KIR genes and their ligands in MERS patient and control groups, KIR3DL1+/Bw4(80I)+, KIR3DL1+/Bw6+, KIR3DL1+/Bw6−, KIR2DS1+/C2+, and KIR3DS+/Bw4(80I)+ were associated with MERS. KIR3DL1+/Bw6− was found in Mo/Mi cases. KIR2DS1+/C2+ and KIR2DS2+/C1+ were found in severe cases. Conclusion: Further investigations are needed to prove the various immune responses of MERS-CoV-infected cells according to variations in the KIR gene and ligand gene. A treatment strategy based on current research on the KIR gene and MERS-CoV will suggest potential treatment targets.

## 1. Introduction

Middle East Respiratory Syndrome (MERS) is a new lower respiratory tract disease with a high mortality rate [1]. It is caused by the high-resolution structures of the trimeric beta coronavirus (CoV) MERS-CoV [2]. The first case of MERS-CoV was reported in Saudi Arabia [3] and has since spread to other countries. From 2013 to April 2018, MERS-CoV infection was confirmed in dromedary camels (*Camelus dromedarius*) in the field. Serological evidence of viral circulation was found in 20 countries and molecular evidence in 13 countries [4].

As of 19 June 2015, South Korea had the highest MERS-CoV infection rate outside of the Arabian Peninsula, with 166 confirmed cases (including 24 deaths). The mean incubation period was 6.7 days and the mean illness duration was 12.6 days. Infection does not appear before symptoms begin. There is an approximately 21% risk of death in all cases of MERS (95% confidence interval: 14–31%) [5]. Hospital-to-hospital transmission started at one hospital in Korea and expanded to 17 hospitals [6]. The outbreak occurred entirely in hospitals and was largely due to failures in infection control and policy. The average age of MERS patients in Korea was 55 years old (the world average is 50 years old). In most countries, including Korea, men were more affected than women. The death rate in Korea was 19.9%, lower than that of global citizens (38.7%) and Saudi Arabians (36.5%). Most Korean patients were infected in hospitals and there was no community spread. Respiratory disease, cancer, and hypertension were the major underlying diseases [7].

Natural killer (NK) cells, large granular cytotoxic lymphocytes that account for 2–18% of human peripheral lymphocytes, play an important role in innate immunity against bacteria, parasites, viruses, and malignant cells [8]. Effector functions of NK cells are regulated by a delicate balance between signals transmitted from activating and inhibitory cell surface receptors [8]. NK cells play an essential role in preventing the formation and progression of leukemia cells [9,10,11].

On the surface of NK cells are different receptors that transmit inhibitory or activating signals [12]. The receptor family is the highly polygenic and polymorphic killer cell immunoglobulin-like receptors (KIRs) with different ligand specificities for human leukocyte antigen class I (HLA-I) molecules [13]. KIRs play a dominant role in the development and functioning of NK cells [14,15]. These receptors are divided into two groups according to the intracellular length of the KIR tail: inhibitory (i) KIRs and activating (a) KIRs. Long-tailed receptors induce inhibition signals via immunoreceptor tyrosine-based inhibitory motifs (ITIMs), while short-tailed receptors transmit activation signals using DAP12 adapter proteins via immunoreceptor tyrosine-based activation motifs (ITAMs) [15,16].

The KIR gene cluster is located on chromosome 19 (19q13.4) and encodes 16 different molecules containing two pseudogenes: KIR2DL1, KIR2DL2/L3, KIR2DL4, KIR2DL5A, KIR2DL5B, KIR2DS1, KIR2DS2, KIR2DS3, KIR2DS4, KIR2DS5, KIR3DL1/S1, KIR3DL2, KIR3DL3, KIR2DP1, and KIR3DP1 [16,17,18,19]. The KIR gene contains two or three extracellular domains (2D or 3D), membrane permeation regions, and long (with inhibitor motifs), or short (with docking sites for molecular activation) tails [17]. Depending on the KIR gene content, there are two KIR haplotypes. “A” haplotypes contain five iKIRs (2DL3, 2DL1, 2DL4, 3DL1, and 3DL2) and one aKIR (2DS4) [20]. In addition to the full-length form (fl), KIR2DS4 has a truncated variant with a 22-bp deletion at exon five (del), which produces a secretory molecule with no known function [21,22]. However, “B” haplotypes mainly possess aKIRs (2DL2, 2DL5, 2DS1, 2DS2, 2DS3, 2DS5, and 3DS1). Both haplotypes have four framework genes (3DL3, 3DP1, 2DL4, and 3DL2) [20]. Individuals can be the AA or Bx (AB or BB) genotype according to their inherited haplotypes. KIR haplotypes are divided into centromeric and telomeric regions via a rearrangement site [17]. Two pseudogenes (KIR2DP1 and KIR3DP1) are contained [23].

HLA-I molecules are the most important ligands for the KIRs. NK cell development and response strongly depend on KIR/HLA-I interaction [24]. HLA-C alleles encode two forms of ligands based on amino acid residues at positions 77 and 80 in their α1 domains. HLA-C1 has serine and aspartic acid, but HLA-C2 has aspartic acid and lysine at these positions, respectively. Therefore, they can interact with different receptors via these residues. HLA-C1 interacts with KIR2DL2/3, and HLA-C2 ligates with KIR2DL1 [15,25]. HLA-B alleles are also classified into Bw4 and Bw6 motifs, and just the former group can bind to KIR3DL1. The affinity of Bw4-bearing allotypes to KIR3DL1 is determined by a dimorphic site at amino acid position 80 (isoleucine or threonine), as HLA-Bw4^Ile80^ binds to the receptor with greater affinity [26,27,28]. The Bw4 motif is also found in HLA-A*23/24/32 alleles, called HLA-A Bw4 [29,30]. Furthermore, HLA-A*03/11 allotypes can interact with KIR3DL2 [31]. Although the extracellular domains of some aKIRs have high sequence homology with some iKIRs (2DS1-2DL1, 2DS2-2DL2, and 3DS1-3DL1 pairs), little information is available about the ligands of aKIRs, and their ligands are mostly unknown except for KIR2DS1 which binds to HLA-C2 [32,33]. Recently, it has been found that some HLA-I ligands can also bind to aKIRs but with lower affinity than the corresponding iKIRs [15].

Downregulation of HLA class I expression due to viral infection mitigates the inhibitory effect on NK cells, allowing NK cells to lyse target cells [34]. Usually, Middle East respiratory syndrome coronavirus (MERS-CoV) and severe acute respiratory syndrome are commonly reported in comparison to COVID-19 (SARS-CoV-2). This was a multi-epitope vaccine against MERS-CoV in silico prediction, bioinformatics analysis, and drug research [35,36,37,38,39,40,41,42]. Cytokine response profiles were analyzed in plasma samples from confirmed MERS-CoV patients [43]. Studies of KIR have been investigated with a focus on SARS-CoV-2 [44,45].

In this study, because no specific KIR association has yet been reported against MERS, we evaluated the association between genetic variation in KIR using polymerase chain reaction with sequence-specific primers (PCR-SSP) and the risk of MERS in South Koreans.

## 2. Materials and Methods

### 2.1. Subjects

#### 2.1.1. MERS Patients

This study comprised 32 patients with MERS admitted to or followed up after recovery at the National Medical Center (NMC) in Seoul, Republic of Korea using the previous study [46]. All patients were confirmed to have MERS-CoV infection by real-time reverse transcription-polymerase chain reaction (PCR) of nasopharyngeal swabs or tracheal aspirates. The patients were classified into two groups depending on the clinical severity during their acute stage of infection. Severe disease (*n* = 16) included fatalities and patients who required mechanical ventilation to relieve respiratory failure. Moderate and mild disease (Mo/Mi) encompassed patients who reported symptoms such as fever, headache, cough, and malaise with or without pulmonary lesions in the absence of pulmonary failure. Demographic characteristics of the patients are shown in Table 1. Peripheral blood was collected from patients at the acute/convalescent phases of infection or after recovery. Peripheral blood mononuclear cells (PBMCs) were isolated from heparinized whole blood by density gradient centrifugation using Ficoll–Paque solution (GE Healthcare, Uppsala, Sweden) and stored in liquid nitrogen until use. The study was approved by the NMC Ethical Committee and written informed consent was obtained from the patients (Institutional Review Board [IRB]: H-1801-086-002, H-1712-085-005).

#### 2.1.2. Control Group

The control group included 200 genetically unrelated healthy Korean adults with no history of MERS and little or no gender distribution (age: 38 ± 8 years; 84 females and 116 males). The control subjects provided written informed consent to participate and mainly comprised students and staff from the Medical College of the Catholic University of Korea and Hematopoietic Stem Cell Transplantation Center. The use of the material was reviewed and approved by the IRB of The Catholic University of Korea with written informed consent obtained for all samples collected (IRB: MC13SISI0126).

### 2.2. Genomic DNA Preparation

We performed this process as previously described [46]. Genomic DNA was isolated from PBMCs using the QIAamp DNA Blood Mini Kit (Qiagen, Hilden, Germany) according to the manufacturer’s instructions. DNA yields and purity were determined by measuring the absorbance at 260 and 280 nm with a NanoDrop 2000 spectrophotometer (Thermo Scientific, Waltham, MA, USA).

### 2.3. Multiplex PCR-SSP for KIR Genotyping and Gel Analysis of PCR Products

A multiplex PCR-SSP method for KIR genotyping has been developed with recently introduced novel primers [47]. Briefly, PCR products were generated by mixing primers for two to four different KIR genes and separating them using an agarose gel. Finally, sixteen KIR genes and the major subtypes of three KIR genes were detected in a set of eight PCR reactions, including the genes encoding human growth hormone (HGH) and adenomatous polyposis (APC) used as an internal amplification control (IC). Amplified PCR products were separated for KIR genotyping using electrophoresis on 3% agarose gel (Bio-rad, Hercules, CA, USA) gel according to a previous study [47].

### 2.4. Statistical Analysis

The observed carrier frequency (OF) of the KIR gene was determined as the number of positive typing responses divided by the total number of individuals typed in the previous study [47]. Estimated gene frequencies (GF) based on the assumption of Hardy–Weinberg equilibrium were calculated using the formula GF = $1-\sqrt{\left(1-OF\right)}$, where OF is the carrier frequency of the KIR gene observed in an individual [47].

For analysis of the case-control study, the previously described analysis methods were used, such as a chi-square test (*χ*^2^), Fisher’s exact test, corrected *p*-values of Bonferroni’s method, 95% confidence intervals (Cis) and odds ratios (ORs) of Haldane’s modification of Woolf’s method [46,48,49,50,51,52,53,54,55,56,57,58]. We defined significant data as *p* < 0.05 and 95% CI ≠ 1 (not containing 1; e.g., 95% CI of a risk effect is 1.01–6.1 or 95% CI of a protective effect is 0.01–0.99).

## 3. Results

### 3.1. Frequencies of KIR Gene Clusters in Patients with MERS-CoV Infection and Controls

The frequency of KIR2DS4D (HLA-C2 group) was higher in the group of all patients with MERS-CoV infection (MERS total) compared with the controls (88 of 200 [44.0%] versus 21 of 32 [65.6%]; *p* = 0.023; OR = 2.4; 95% CI 1.1–5.3). KIR3DP1F frequencies were higher in the group of all patients with MERS-CoV infection (MERS total) compared with the controls (19 of 200 [9.5%] versus 7 of 32 [21.9%]; *p* = 0.039; OR = 2.7; 95% CI 1.02–7.0). KIR2DL1 (HLA-C2 group), KIR2DP1, and KIR3DP1D were lower in Mo/Mi cases (each 200 of 200 [100%] versus 15 of 16 [93.8%]; *p* = 0.018; OR = 0.04; 95% CI 0.003–0.5). KIR2DL2, HLA-C1 group, was higher in severe cases of MERS-CoV infection compared with the control group (25 of 200 [12.5%] versus 5 of 16 [31.3%]; *p* = 0.040; OR = 3.2; 95% CI 1.02–9.9). KIR2DS2 and KIR3DP1F were higher (31 of 200 [15.5%] versus 6 of 16 [37.5%]; *p* = 0.025; OR = 3.3; 95% CI 1.1–9.7, 19 of 200 [9.5%] versus 5 of 16 [31.3%]; *p* = 0.017; OR = 4.3; 95% CI 1.4–13.8) (Table 2).

### 3.2. The Association between KIR Genes and Their Ligands in MERS Patients versus Control Groups

When we investigated the association between KIR genes and their ligands in MERS patients versus control groups, we found the following results: KIR3DL1+/Bw4(80I)+ (68 of 200 [34.0%] versus 17 of 32 [53.1%]; *p* = 0.037; OR = 2.2; 95% CI 1.04–4.7), KIR3DL1+/Bw6+ (143 of 200 [71.5%] versus 17 of 32 [53.1%]; *p* = 0.037; OR = 0.5; 95% CI 0.2–0.97), KIR3DL1+/Bw6− (40 of 200 [20.0%] versus 13 of 32 [40.6%]; *p* = 0.010; OR = 2.7; 95% CI 1.2–6.0), KIR2DS1+/C2+ (23 of 200 [11.5%] versus 8 of 32 [25.0%]; *p* = 0.037; OR = 2.6; 95% CI 1.03–6.4), and KIR3DS+/Bw4(80I)+ (29 of 200 [14.5%] versus 10 of 32 [31.3%]; *p* = 0.019; OR = 2.7; 95% CI 1.2–6.2) were associated with the group of all patients with MERS-CoV infection (MERS total). KIR3DL1+/Bw6− was also found more often in Mo/Mi cases (40 of 200 [20.0%] versus 7 of 32 [43.8%]; *p* = 0.019; OR = 2.7; 95% CI 1.2–6.2). KIR2DS1+/C2+ (23 of 200 [11.5%] versus 5 of 32 [31.3%]; *p* = 0.040; OR = 3.5; 95% CI 1.1–11.0) and KIR2DS2+/C1+ (31 of 200 [15.5%] versus 6 of 32 [37.5%]; *p* = 0.025; OR = 3.3; 95% CI 1.1–8.9) were both found more often in severe cases (Table 3).

We updated the previous study [46] as the association analysis of the HLA-A, -B, and -C genes between the added control group (*n* = 200) and the group of patients with MERS (*n* = 32) (Tables S1–S5). At the allele-level, the HLA-C*01:02 allele frequency was lower in the Mo/Mi cases of MERS-CoV infection compared with the controls but did not show statistical significance by Bonferroni’s correction (72 of 200 [36.0%] versus 1 of 16 [6.3%]; *p* = 0.009; OR = 0.1; 95% CI 0.015–0.92; *p_c_* = NS). Genotypes C1C1, C1C2, and C2C2 showed no significant association with MERS-CoV infection (Table S5).

### 3.3. Association of KIR Haplotype and Genotype with MERS Risk

When we classified genomic data into the AA and Bx haplotypes, AA genotype ID one haplotype was lower in the group of all patients with MERS-CoV infection (MERS total) compared with the controls (111 of 200 [55.5%] versus 11 of 32 [34.4%]; *p* = 0.026; OR = 0.4; 95% CI 0.19–0.92). However, Bx genotype ID 4 haplotype was higher in severe cases of MERS-CoV infection compared with the controls (9 of 200 [4.5%] versus 3 of 16 [18.8%]; *p* = 0.042; OR = 4.9; 95% CI 1.2–20.3). Bx genotype ID 7 and 36 showed risk effects in severe cases and Bx genotype ID 72 showed risk effects in Mo/Mi cases of MERS-CoV infection compared with the controls (each 0 of 200 [0.0%] versus 1 of 16 [6.3%]; *p* = 0.018; OR = 25.1; 95% CI 2.2–292.3) (Table S6).

## 4. Discussion

### 4.1. KIR Genes

**Figure 1:**
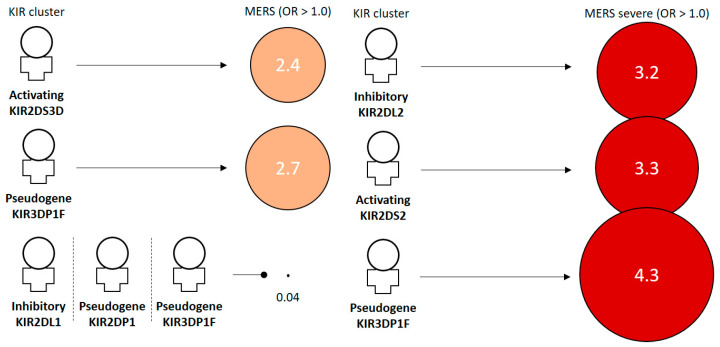
Risk effects of MERS stages against KIR clusters. OR, odd ratio; OR > 1.0, risk effect; OR < 1.0, protective effect.

**Figure 2:**
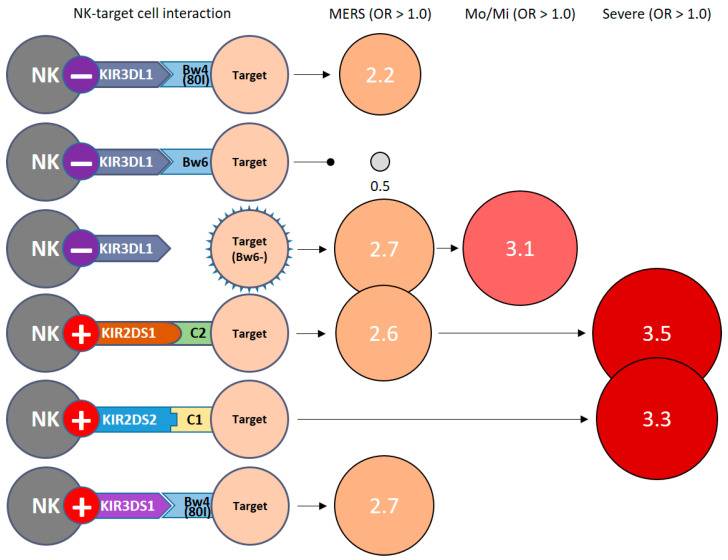
Risk effects of MERS stages against NK-target cell interactions. OR, odd ratio; OR > 1.0, risk effect; OR < 1.0, protective effect; Mo/Mi, moderate and mild; −, inhibitory KIRs; +, activating KIRs.

We investigated the association of KIR gene clusters with MERS-CoV in 32 patients with MERS. We summarized the risk effects of MERS stages against KIR clusters and NK-target cell interactions (Figure 1 and Figure 2) based on Table 1 and Table 2. To date, no research has been conducted on the association of KIR genes with MERS-CoV infection. We were able to discuss the current controversial COVID-19 situation indirectly. Moreover, similar to SARS and MERS [59], this extrapolation can be made because three human beta-coronaviruses share common features: (i) similar genomes (80% homogeneity between SARS-CoV-2 and SARS-CoV; and 50% with MERS-CoV); (ii) high transmission rates and similar transmission modes; and (iii) severe clinical infection spectrum with development of lung damage and cytokine storms [60,61,62]. Reduced lymphocytes, particularly CD4+CD8+ T lymphocytes, have been found in patients with early-stage COVID-19. Decreased lymphocytes are also important predictors of disease severity [63,64]. In the recent SARS-CoV-2 (COVID-19) association study, it has been reported that an individualized constellation of killer cell immunoglobulin-like receptors and their cognate HLA class I ligands, which control natural killer cell antiviral immunity, causes COVID-19 [65].

Frequencies of inhibitor KIR2DL1 (C2) are lower in Mo/Mi cases of MERS-CoV infection compared to the control group (*p* = 0.018; OR = 0.04) (Table 2). In the COVID-19 study, the authors characterized the expansion of the NK subset with low cell degradation activity in relation to the immunological scenario of severe COVID-19 infection, enhanced expression of 2DL1 inhibitory receptors, and C2 ligand (KIR2DL1-C2) recovered from mild/moderate/severe SARS-CoV-2 infection in 30 patients suffering from neurological conditions. The control group had only 10 cases of SARS-CoV-2 infections [66].

A recent COVID-19 study suggested that KIR2DL2/HLA-C1C1 gene pairs could be a risk factor for SARS-CoV-2 infection [67]. When comparing 158 mild COVID-19 patients, 99 severely ill patients, and 98 healthy controls, KIR2DL2, KIR2DS3, HLA-C1C1, and KIR2DL2/HLA-C1 pairing frequencies were significantly higher in the COVID-19 patients than in healthy controls. The frequency of KIR2DL3+KIR2DL2-/HLA-C1+ other+ was lower in COVID-19 patients than in healthy individuals. These results suggested that the protective effect of KIR2DL3 on SARS-CoV-2 infection is associated with the absence of the KIR2DL2 gene. No correlation was found between the frequency of these genes and the outbreak of COVID-19 [67]. In this study, clusters of KIR2DL1/2/3 and 2DS1/2 genes also showed an independent association with HLA-C alleles (Table S5).

### 4.2. HLA Class I Ligands

Inhibitor KIR3DL1+/HLA-Bw4(80I)+ and KIR3DL1+/HLA-Bw4(80I)- showed conflicting results among patients with MERS-CoV infection (*p* = 0.037; OR = 2.2and *p* = 0.037; OR = 0.5, respectively). A recent study investigated the effect of HLA and KIR genotypes and HLA-KIR combinations on COVID-19 outcomes and found that the peptide affinity of HLA alleles was not correlated with COVID-19 severity [68]. Predicted bad binders for SARS-CoV-2 peptides encode KIR ligands containing Bw4 and C1 (introduced by B*46:01) which have small F pockets and cannot accommodate SARS-CoV-2. It belongs to the HLA-B subtype cytotoxic T lymphocytes (CTL) epitope. However, HLA-Bw4 weak binders were beneficial for COVID-19 results, and individuals without HLA-Bw4 motifs had a higher risk of developing serious diseases due to COVID-19. The presence of a combination of HLA-Bw4 and KIR3DL1 lowered the risk of developing severe COVID-19 by 58.8% (OR = 0.412, 95% CI = 0.165–0.904, *p* = 0.02). This suggests that HLA-Bw4 alleles that impair the ability to load SARS-CoV-2 peptides will be targeted for NK-mediated destruction [68]. Ninety-six Sicilian patients were tested for KIR and HLA ligands, including 56 chronic hepatitis B (CHB) patients who were human immunodeficiency virus-1 (HIV) positive and 92 SARS-CoV-2 positive Sicilian patients [69]. Inhibitive KIR3DL1 gene and KIR3DL1/HLA-B Bw4 pairs were more common in individual CHB patients. The frequency of HLA-C2 was higher in CHB compared to other groups. The HLA-C1 ligand did not appear to contribute to CHB progression but had a significantly higher frequency in COVID-19 and HIV-positive than in healthy controls [69]. Additionally, we investigated the influence on specific locations, KIR3DL1+/HLA-Bw4(80I)+, and in all patients with MERS-CoV infection, so we suggest that synergistic reactions of CTLs and NK cells can efficiently control MERS-CoV infection and replication, and that NK cell-mediated anti-MERS-CoV immune responses are primarily involved in severe infections according to the study of SARS-CoV-2 (Table 3).

### 4.3. MICA Molecules

As an important ligand of human NKG2D, as well as another activating cell surface receptor expressed on NK cells and some T-cell subsets, the MHC class I chain-related gene A (*MICA*) molecule is expressed on several tumors, especially on epithelium-derived cancer cells [70]. Ligand binding of *MICA* to the NKG2D receptor mediates innate immune response by stimulating NK cells and γδT cells. Tumor cells expressing high levels of membrane *MICA* molecule and other NKG2D ligands are rejected by NK cells and CD8aβT cells and stimulate antitumor activity, while reduced expression of membrane *MICA* molecule, as well as increased soluble *MICA* molecules in the serum, leads to inactivation of NKG2D-mediated antitumor response [71,72,73,74,75]. It is assumed that the reduction in membrane *MICA* molecules containing MICA*002:01 and A9 alleles by release or shedding from the cell surface would potentially reduce the immunogenetic signals of tumor cells, and then the tumor cells would become less detectable by NK and T cells. Finding that strong linkage disequilibrium (LD) between HLA-B58 and *MICA*-STR A9 existed, the haplotype HLA-B58-*MICA*-STR A9 did not impose a higher risk for leukemia [76]. Similar results have been reported in many autoimmune diseases [77,78], including the previous study on the association between MICA/B and leukemia [51], and the reason why the *MICA* gene seemed to be associated with diseases independent of the HLA gene remains unknown. Further work needs to be completed to functionally resolve the possible association between other HLA-MICA haplotypes and MERS-CoV infection.

### 4.4. Limitation

The number of samples is very limited at 32. However, considering the scarcity of samples, it can be assumed that collecting samples would not have been easy, so it is reasonable to place meaning on the samples themselves rather than the number of samples. Further work needs to be completed to functionally resolve the possible association between KIR gene clusters and MERS-CoV infection.

## 5. Conclusions

In conclusion, it remains unclear why some patients suffer from MERS-CoV. Genetic variations of the KIR genes and their ligand genes may affect MERS-CoV infection in Koreans. Further investigations are needed to demonstrate the different immune responses of NK cells against MERS-CoV infection according to genetic variation in KIR genes and their ligand genes. This evidence will help inform NK cell-based immunotherapy for patients with MERS-CoV infection. Current research in KIR gene clusters and treatment strategies based on their association with MERS-CoV can provide potential treatment targets.

## Figures and Tables

**Table 1:** Characteristics of patients with MERS [46].

	Group (Clinical Severity)		Total
	Mo/Mi	Severe	
No. of patients (%)	16 (50)	16 (50)	32
Male/female (*n*)	8/8	13/3	21/11
Age (year), mean ± SD	50 ± 21	58 ± 15	54 ± 19
Underlying disease, *n* (%)			
Diabetes	1 (6.3)	3 (18.8)	4 (12.5)
Chronic renal disease	1 (6.3)	2 (12.5)	3 (9.4)
Chronic cardiac disease	1 (6.3)	4 (25.0)	5 (15.6)
Chronic pulmonary disease	0 (0)	1 (6.3)	1 (3.1)
Hypertension	2 (12.5)	7 (43.8)	9 (28.1)
Chronic liver disease	1 (6.3)	0 (0)	1 (3.1)
Solid tumor	2 (12.5)	2 (12.5)	4 (12.5)
Obesity	0 (0)	1 (6.3)	1 (3.1)

MERS, middle east respiratory syndrome; Mo/Mi, Moderate and Mild.

**Table 2:** Genetic influence of KIR genes in MERS patients.

Function	Gene Clusters	Controls		MERS Total		Mo/Mi Cases		Severe Cases	
		*n* = 200 (%)		*n* = 32 (%)		*n* = 16 (%)		*n* = 16 (%)	
Inhibitory	KIR2DL1	200	(100.0)	31	(96.9)	15	(93.8) ^c^	16	(100.0)
	KIR2DL2	25	(12.5)	8	(25.0)	3	(18.8)	5	(31.3) ^f^
	KIR2DL3	199	(99.5)	31	(96.9)	15	(93.8)	16	(100.0)
	KIR2DL4	200	(100.0)	32	(100.0)	16	(100.0)	16	(100.0)
	KIR2DL5	74	(37.0)	17	(53.1)	9	(56.3)	8	(50.0)
	KIR2DL5A	69	(34.5)	16	(50.0)	9	(56.3)	7	(43.8)
	KIR2DL5B	14	(7.0)	2	(6.3)	1	(6.3)	1	(6.3)
	KIR3DL1	183	(91.5)	30	(93.8)	15	(93.8)	15	(93.8)
	KIR3DL2	200	(100.0)	32	(100.0)	16	(100.0)	16	(100.0)
	KIR3DL3	200	(100.0)	32	(100.0)	16	(100.0)	16	(100.0)
Activating	KIR2DS1	72	(36.0)	16	(50.0)	9	(56.3)	7	(43.8)
	KIR2DS2	31	(15.5)	9	(28.1)	3	(18.8)	6	(37.5) ^g^
	KIR2DS3	37	(18.5)	9	(28.1)	4	(25.0)	5	(31.3)
	KIR2DS4	183	(91.5)	31	(96.9)	15	(93.8)	16	(100.0)
	KIR2DS4D	88	(44.0)	21	(65.6) ^a^	11	(68.8)	10	(62.5)
	KIR2DS4F	146	(73.0)	23	(71.9)	10	(62.5)	13	(81.3)
	KIR2DS5	48	(24.0)	9	(28.1)	6	(37.5)	3	(18.8)
	KIR3DS1	73	(36.5)	16	(50.0)	9	(56.3)	7	(43.8)
Pseudogene	KIR2DP1	200	(100.0)	31	(96.9)	15	(93.8) ^d^	16	(100.0)
	KIR3DP1	200	(100.0)	32	(100.0)	16	(100.0)	16	(100.0)
	KIR3DP1D	200	(100.0)	31	(96.9)	15	(93.8) ^e^	16	(100.0)
	KIR3DP1F	19	(9.5)	7	(21.9) ^b^	2	(12.5)	5	(31.3) ^h^

Controls versus MERS total: ^a^ OR = 2.4 (1.1–5.3), *p* = 0.023; ^b^ OR = 2.7 (1.02–7.0), *p* = 0.039. Controls versus Mo/Mi cases: ^c^ OR = 0.04 (0.003–0.5), *p* = 0.018; ^d^ OR = 0.04 (0.003–0.5), *p* = 0.018; ^e^ OR = 0.04 (0.003–0.5), *p* = 0.018. Controls versus Severe cases: ^f^ OR = 3.2 (1.02–9.9), *p* = 0.037; ^g^ OR = 3.3 (1.1–9.7), *p* = 0.025; ^h^ OR = 4.3 (1.4–13.8), *p* = 0.017.

**Table 3:** The association of KIR genes and their ligands between MERS patients and control groups.

KIR Genes	Ligand Genes (HLA)	Controls		MERS Total		Mo/Mi Cases		Severe Cases	
		*n* = 200 (%)		*n* = 32 (%)		*n* = 16 (%)		*n* = 16 (%)	
KIR2DL1+	C2+	61	(30.5)	12	(37.5)	6	(37.5)	6	(37.5)
	C2−	139	(69.5)	19	(59.4)	9	(56.3)	10	(62.5)
KIR2DL2+	C1+	25	(12.5)	8	(25.0)	3	(18.8)	5	(31.3)
	C1−	0	(0.0)	0	(0.0)	0	(0.0)	0	(0.0)
KIR2DL3+	C1+	192	(96.0)	31	(96.9)	15	(93.8)	16	(100.0)
	C1−	7	(3.5)	0	(0.0)	0	(0.0)	0	(0.0)
KIR3DL1+	Bw4+	133	(66.5)	25	(78.1)	11	(68.8)	14	(87.5)
	BW4−	50	(25.0)	5	(15.6)	4	(25.0)	1	(6.3)
	Bw4 (80I)+	68	(34.0)	17	(53.1) ^a^	8	(50.0)	9	(56.3)
	Bw4 (80I)−	115	(57.5)	13	(40.6)	7	(43.8)	6	(37.5)
	Bw4 (80T)+	33	(16.5)	5	(15.6)	1	(6.3)	4	(25.0)
	Bw4 (80T)−	150	(75.0)	25	(78.1)	14	(87.5)	11	(68.8)
	Bw6+	143	(71.5)	17	(53.1) ^b^	8	(50.0)	9	(56.3)
	Bw6−	40	(20.0)	13	(40.6) ^c^	7	(43.8) ^f^	6	(37.5)
KIR3DL2+	A3+ or A11+	49	(24.5)	6	(18.8)	3	(18.8)	3	(18.8)
	A3− and A11−	151	(75.5)	26	(81.3)	13	(81.3)	13	(81.3)
KIR2DS1+	C2+	23	(11.5)	8	(25.0) ^d^	3	(18.8)	5	(31.3) ^g^
	C2−	49	(24.5)	8	(25.0)	6	(37.5)	2	(12.5)
KIR2DS2+	C1+	31	(15.5)	9	(28.1)	3	(18.8)	6	(37.5) ^h^
	C1−	0	(0.0)	0	(0.0)	0	(0.0)	0	(0.0)
KIR3DS1+	Bw4+	56	(28.0)	13	(40.6)	7	(43.8)	6	(37.5)
	Bw4−	17	(8.5)	3	(9.4)	2	(12.5)	1	(6.3)
	Bw4 (80I)+	29	(14.5)	10	(31.3) ^e^	5	(31.3)	5	(31.3)
	Bw4 (80I)−	44	(22.0)	6	(18.8)	4	(25.0)	2	(12.5)
	Bw4 (80T)+	13	(6.5)	4	(12.5)	1	(6.3)	3	(18.8)
	Bw4 (80T)−	60	(30.0)	12	(37.5)	8	(50.0)	4	(25.0)

Controls versus MERS total: ^a^ OR = 2.2 (1.04–4.7), *p* = 0.037; ^b^ OR = 0.5 (0.2–0.97), *p* = 0.037; ^c^ OR = 2.7 (1.2–6.0), *p* = 0.010; ^d^ OR = 2.6 (1.03–6.4), *p* = 0.037; ^e^ OR = 2.7 (1.2–6.2), *p* = 0.019. Controls versus Mo/Mi cases: ^f^ OR = 3.1 (1.1–8.9), *p* = 0.027. Controls versus Severe cases: ^g^ OR = 3.5 (1.1–11.0), *p* = 0.040; ^h^ OR = 3.3 (1.1–9.7), *p* = 0.025. C1 group: Ser77Asn80 allele among HLA-C alleles. C2 group: Asn77Lys80 allele among HLA-C alleles.

## Data Availability

Data are available on request due to privacy/ethical restrictions.

## Supplementary Materials

The following supporting information can be downloaded at: https://www.mdpi.com/article/10.3390/jcm13010258/s1, Table S1: Genetic influence of HLA-A in MERS patients. Table S2: Genetic influence of HLA-B in MERS patients. Table S3: Genetic influence of HLA-Bw4 in MERS patients; Table S4: Genetic influence of HLA-Bw6 in MERS patients. Table S5: Genetic influence of HLA-C in MERS patients. Table S6. Genetic influence of KIR haplotypes in MERS patients.
