# Supplementary material for: Association of KIR Genes with Middle East Respiratory Syndrome Coronavirus Infection in South Koreans
Source: J Clin Med. 2024 Jan 2;13(1):258. doi: 10.3390/jcm13010258 (PMC10779705; doi:10.3390/jcm13010258)
Supplement: Supplementary file 1 [file jcm-13-00258-s001.zip › jcm-2710518-supplementary.pdf]

Table S1. Genetic influence of HLA-A in MERS patients

		Controls	MERS total					Mo/Mi cases					Severe cases				
Group	Alleles	n = 200 (%)	n = 32 (%)	χ^2	p-value	P_c	OR	n = 16 (%)	χ^2	p-value	P_c	OR	n = 16 (%)	χ^2	p-value	P_c	OR
A1	01:01	5 (2.5)	2 (6.3)	1.326	0.250	9.484	NA	1 (6.3)	0.771	0.380	14.431	NA	1 (6.3)	0.771	0.380	14.431	NA
A2	02:01	73 (36.5)	8 (25.0)	1.605	0.205	7.795	NA	4 (25.0)	0.854	0.355	13.505	NA	4 (25.0)	0.854	0.355	13.505	NA
A2	02:04	0 (0.0)	0 (0.0)	NA	NA	NA	NA	0 (0.0)	NA	NA	NA	NA	0 (0.0)	NA	NA	NA	NA
A2	02:05	0 (0.0)	0 (0.0)	NA	NA	NA	NA	0 (0.0)	NA	NA	NA	NA	0 (0.0)	NA	NA	NA	NA
A2	02:06	35 (17.5)	5 (15.6)	0.068	0.794	30.184	NA	3 (18.8)	0.016	0.899	34.179	NA	2 (12.5)	0.261	0.609	23.161	NA
A2	02:07	16 (8.0)	3 (9.4)	0.069	0.792	30.106	NA	1 (6.3)	0.063	0.802	30.494	NA	2 (12.5)	0.393	0.531	20.173	NA
A2	02:08	0 (0.0)	0 (0.0)	NA	NA	NA	NA	0 (0.0)	NA	NA	NA	NA	0 (0.0)	NA	NA	NA	NA
A2	02:09	0 (0.0)	0 (0.0)	NA	NA	NA	NA	0 (0.0)	NA	NA	NA	NA	0 (0.0)	NA	NA	NA	NA
A2	02:11	0 (0.0)	0 (0.0)	NA	NA	NA	NA	0 (0.0)	NA	NA	NA	NA	0 (0.0)	NA	NA	NA	NA
A2	02:17	0 (0.0)	0 (0.0)	NA	NA	NA	NA	0 (0.0)	NA	NA	NA	NA	0 (0.0)	NA	NA	NA	NA
A203	02:03	7 (3.5)	0 (0.0)	1.155	0.283	10.736	NA	0 (0.0)	0.579	0.447	16.978	NA	0 (0.0)	0.579	0.447	16.978	NA
A210	02:10	0 (0.0)	0 (0.0)	NA	NA	NA	NA	0 (0.0)	NA	NA	NA	NA	0 (0.0)	NA	NA	NA	NA
A3	03:01	7 (3.5)	1 (3.1)	0.012	0.914	34.734	NA	0 (0.0)	0.579	0.447	16.978	NA	1 (6.3)	0.314	0.575	21.856	NA
A3	03:02	0 (0.0)	0 (0.0)	NA	NA	NA	NA	0 (0.0)	NA	NA	NA	NA	0 (0.0)	NA	NA	NA	NA
A11	11:01	42 (21.0)	5 (15.6)	0.493	0.482	18.333	NA	3 (18.8)	0.045	0.831	31.583	NA	2 (12.5)	0.660	0.417	15.831	NA
A11	11:02	1 (0.5)	1 (3.1)	2.224	0.136	5.163	NA	1 (6.3)	5.339	**0.021**	0.792	NA	0 (0.0)	0.080	0.777	29.518	NA
A11	11:05	0 (0.0)	0 (0.0)	NA	NA	NA	NA	0 (0.0)	NA	NA	NA	NA	0 (0.0)	NA	NA	NA	NA
A11	11:03	1 (0.5)	0 (0.0)	0.161	0.689	26.164	NA	0 (0.0)	0.080	0.777	29.518	NA	0 (0.0)	0.080	0.777	29.518	NA
A23(9)	23:01	0 (0.0)	0 (0.0)	NA	NA	NA	NA	0 (0.0)	NA	NA	NA	NA	0 (0.0)	NA	NA	NA	NA
A24(9)	24:02	75 (37.5)	14 (43.8)	0.456	0.500	18.986	NA	7 (43.8)	0.246	0.620	23.564	NA	7 (43.8)	0.246	0.620	23.564	NA
A2403	24:03	0 (0.0)	0 (0.0)	NA	NA	NA	NA	0 (0.0)	NA	NA	NA	NA	0 (0.0)	NA	NA	NA	NA
A24(9)	24:07	0 (0.0)	0 (0.0)	NA	NA	NA	NA	0 (0.0)	NA	NA	NA	NA	0 (0.0)	NA	NA	NA	NA
A26(10)	26:01	12 (6.0)	2 (6.3)	0.003	0.956	36.329	NA	2 (12.5)	1.033	0.310	11.762	NA	0 (0.0)	1.016	0.313	11.908	NA
A26(10)	26:02	18 (9.0)	0 (0.0)	3.122	0.077	2.935	NA	0 (0.0)	1.571	0.210	7.983	NA	0 (0.0)	1.571	0.210	7.983	NA
A26(10)	26:03	1 (0.5)	0 (0.0)	0.161	0.689	26.164	NA	0 (0.0)	0.080	0.777	29.518	NA	0 (0.0)	0.080	0.777	29.518	NA
A29(19)	29:01	1 (0.5)	1 (3.1)	2.224	0.136	5.163	NA	1 (6.3)	5.339	**0.021**	0.792	NA	0 (0.0)	0.080	0.777	29.518	NA
A29(19)	29:02	0 (0.0)	0 (0.0)	NA	NA	NA	NA	0 (0.0)	NA	NA	NA	NA	0 (0.0)	NA	NA	NA	NA
A30(19)	30:01	12 (6.0)	4 (12.5)	1.815	0.178	6.760	NA	1 (6.3)	0.002	0.968	36.774	NA	3 (18.8)	3.727	0.054	2.035	NA
A30(19)	30:04	9 (4.5)	0 (0.0)	1.498	0.221	8.397	NA	0 (0.0)	0.751	0.386	14.670	NA	0 (0.0)	0.751	0.386	14.670	NA
A31(19)	31:01	21 (10.5)	4 (12.5)	0.115	0.735	27.922	NA	1 (6.3)	0.293	0.589	22.367	NA	3 (18.8)	1.021	0.312	11.867	NA
A32(19)	32:01	1 (0.5)	0 (0.0)	0.161	0.689	26.164	NA	0 (0.0)	0.080	0.777	29.518	NA	0 (0.0)	0.080	0.777	29.518	NA
A33(19)	33:01	0 (0.0)	0 (0.0)	NA	NA	NA	NA	0 (0.0)	NA	NA	NA	NA	0 (0.0)	NA	NA	NA	NA
A33(19)	33:03	53 (26.5)	8 (25.0)	0.032	0.858	32.603	NA	5 (31.3)	0.170	0.680	25.838	NA	3 (18.8)	0.463	0.496	18.851	NA
A33(19)	33:25	0 (0.0)	0 (0.0)	NA	NA	NA	NA	0 (0.0)	NA	NA	NA	NA	0 (0.0)	NA	NA	NA	NA
A6601	66:01	0 (0.0)	0 (0.0)	NA	NA	NA	NA	0 (0.0)	NA	NA	NA	NA	0 (0.0)	NA	NA	NA	NA
A68(28)	68:01	0 (0.0)	0 (0.0)	NA	NA	NA	NA	0 (0.0)	NA	NA	NA	NA	0 (0.0)	NA	NA	NA	NA
A68(28)	68:02	0 (0.0)	0 (0.0)	NA	NA	NA	NA	0 (0.0)	NA	NA	NA	NA	0 (0.0)	NA	NA	NA	NA
A74(19)	74:01	0 (0.0)	0 (0.0)	NA	NA	NA	NA	0 (0.0)	NA	NA	NA	NA	0 (0.0)	NA	NA	NA	NA
A3A3		0 (0.0)	0 (0.0)	NA	NA	NA	NA	0 (0.0)	NA	NA	NA	NA	0 (0.0)	NA	NA	NA	NA
A3A11		0 (0.0)	0 (0.0)	NA	NA	NA	NA	0 (0.0)	NA	NA	NA	NA	0 (0.0)	NA	NA	NA	NA
A11A11		2 (1.0)	1 (3.1)	0.976	0.323	0.970	NA	1 (6.3)	2.981	0.084	0.253	NA	0 (0.0)	0.161	0.688	2.063	NA

P_c, Bonferroni's correction; NA, not applicable

Table S2. Genetic influence of HLA-B in MERS patients

		Controls	MERS total					Mo/Mi cases					Severe cases				
Locus	Alleles	n = 200 (%)	n = 32 (%)	χ^2	p-value	P_c	OR	n = 16 (%)	χ^2	p-value	P_c	OR	n = 16 (%)	χ^2	p-value	P_c	OR
B7	07:02	18 (9.0)	2 (6.3)	0.265	0.607	47.332	NA	1 (6.3)	0.140	0.709	55.273	NA	1 (6.3)	0.140	0.709	55.273	NA
B7	07:05	2 (1.0)	1 (3.1)	0.976	0.323	25.210	NA	1 (6.3)	2.981	0.084	6.570	NA	0 (0.0)	0.161	0.688	53.647	NA
B8	08:01	2 (1.0)	0 (0.0)	0.323	0.570	44.455	NA	0 (0.0)	0.161	0.688	53.647	NA	0 (0.0)	0.161	0.688	53.647	NA
B13	13:01	9 (4.5)	1 (3.1)	0.126	0.722	56.327	NA	1 (6.3)	0.103	0.749	58.387	NA	0 (0.0)	0.751	0.386	30.113	NA
B13	13:02	14 (7.0)	2 (6.3)	0.024	0.876	68.364	NA	0 (0.0)	1.198	0.274	21.356	NA	2 (12.5)	0.653	0.419	32.674	NA
B64(14)	14:01	8 (4.0)	0 (0.0)	1.326	0.250	19.466	NA	0 (0.0)	0.665	0.415	32.365	NA	0 (0.0)	0.665	0.415	32.365	NA
B65(14)	14:02	0 (0.0)	0 (0.0)	NA	NA	NA	NA	0 (0.0)	NA	NA	NA	NA	0 (0.0)	NA	NA	NA	NA
B62(15)	15:01	34 (17.0)	4 (12.5)	0.408	0.523	40.799	NA	3 (18.8)	0.032	0.858	66.933	NA	1 (6.3)	1.261	0.261	20.396	NA
B75(15)	15:02	1 (0.5)	0 (0.0)	0.161	0.689	53.705	NA	0 (0.0)	0.080	0.777	60.590	NA	0 (0.0)	0.080	0.777	60.590	NA
B72(70)	15:03	0 (0.0)	0 (0.0)	NA	NA	NA	NA	0 (0.0)	NA	NA	NA	NA	0 (0.0)	NA	NA	NA	NA
B62(15)	15:07	3 (1.5)	1 (3.1)	0.430	0.512	39.938	NA	0 (0.0)	0.243	0.622	48.498	NA	1 (6.3)	1.839	0.175	13.655	NA
B75(15)	15:08	0 (0.0)	0 (0.0)	NA	NA	NA	NA	0 (0.0)	NA	NA	NA	NA	0 (0.0)	NA	NA	NA	NA
B71(70)	15:10	0 (0.0)	0 (0.0)	NA	NA	NA	NA	0 (0.0)	NA	NA	NA	NA	0 (0.0)	NA	NA	NA	NA
B75(15)	15:11	3 (1.5)	3 (9.4)	6.791	**0.009**	0.715	NA	1 (6.3)	1.839	0.175	13.655	NA	2 (12.5)	7.928	**0.005**	0.380	NA
B76(15)	15:12	0 (0.0)	0 (0.0)	NA	NA	NA	NA	0 (0.0)	NA	NA	NA	NA	0 (0.0)	NA	NA	NA	NA
B63(15)	15:17	0 (0.0)	0 (0.0)	NA	NA	NA	NA	0 (0.0)	NA	NA	NA	NA	0 (0.0)	NA	NA	NA	NA
B71(70)	15:18	8 (4.0)	0 (0.0)	1.326	0.250	19.466	NA	0 (0.0)	0.665	0.415	32.365	NA	0 (0.0)	0.665	0.415	32.365	NA
B62(15)	15:25	0 (0.0)	0 (0.0)	NA	NA	NA	NA	0 (0.0)	NA	NA	NA	NA	0 (0.0)	NA	NA	NA	NA
B62(15)	15:27	0 (0.0)	0 (0.0)	NA	NA	NA	NA	0 (0.0)	NA	NA	NA	NA	0 (0.0)	NA	NA	NA	NA
B18	18:01	0 (0.0)	0 (0.0)	NA	NA	NA	NA	0 (0.0)	NA	NA	NA	NA	0 (0.0)	NA	NA	NA	NA
B18	18:03	0 (0.0)	0 (0.0)	NA	NA	NA	NA	0 (0.0)	NA	NA	NA	NA	0 (0.0)	NA	NA	NA	NA
B18	18:04	0 (0.0)	0 (0.0)	NA	NA	NA	NA	0 (0.0)	NA	NA	NA	NA	0 (0.0)	NA	NA	NA	NA
B27	27:02	0 (0.0)	0 (0.0)	NA	NA	NA	NA	0 (0.0)	NA	NA	NA	NA	0 (0.0)	NA	NA	NA	NA
B27	27:03	0 (0.0)	0 (0.0)	NA	NA	NA	NA	0 (0.0)	NA	NA	NA	NA	0 (0.0)	NA	NA	NA	NA
B27	27:04	1 (0.5)	1 (3.1)	2.224	0.136	10.597	NA	1 (6.3)	5.339	**0.021**	1.626	NA	0 (0.0)	0.080	0.777	60.590	NA
B27	27:05	15 (7.5)	0 (0.0)	2.566	0.109	8.517	NA	0 (0.0)	1.290	0.256	19.978	NA	0 (0.0)	1.290	0.256	19.978	NA
B27	27:06	0 (0.0)	0 (0.0)	NA	NA	NA	NA	0 (0.0)	NA	NA	NA	NA	0 (0.0)	NA	NA	NA	NA
B27	27:07	0 (0.0)	0 (0.0)	NA	NA	NA	NA	0 (0.0)	NA	NA	NA	NA	0 (0.0)	NA	NA	NA	NA
B35	35:01	18 (9.0)	3 (9.4)	0.005	0.945	73.731	NA	2 (12.5)	0.216	0.642	50.084	NA	1 (6.3)	0.140	0.709	55.273	NA
B35	35:02	0 (0.0)	0 (0.0)	NA	NA	NA	NA	0 (0.0)	NA	NA	NA	NA	0 (0.0)	NA	NA	NA	NA
B35	35:03	1 (0.5)	0 (0.0)	0.161	0.689	53.705	NA	0 (0.0)	0.080	0.777	60.590	NA	0 (0.0)	0.080	0.777	60.590	NA
B35	35:05	0 (0.0)	0 (0.0)	NA	NA	NA	NA	0 (0.0)	NA	NA	NA	NA	0 (0.0)	NA	NA	NA	NA
B35	35:08	0 (0.0)	0 (0.0)	NA	NA	NA	NA	0 (0.0)	NA	NA	NA	NA	0 (0.0)	NA	NA	NA	NA
B7	35:21	0 (0.0)	0 (0.0)	NA	NA	NA	NA	0 (0.0)	NA	NA	NA	NA	0 (0.0)	NA	NA	NA	NA
B37	37:01	4 (2.0)	2 (6.3)	1.978	0.160	12.450	NA	1 (6.3)	1.183	0.277	21.580	NA	1 (6.3)	1.183	0.277	21.580	NA
B38(16)	38:01	0 (0.0)	0 (0.0)	NA	NA	NA	NA	0 (0.0)	NA	NA	NA	NA	0 (0.0)	NA	NA	NA	NA
B27	38:02	7 (3.5)	1 (3.1)	0.012	0.914	71.295	NA	1 (6.3)	0.314	0.575	44.862	NA	0 (0.0)	0.579	0.447	34.850	NA
B3901	39:01	7 (3.5)	1 (3.1)	0.012	0.914	71.295	NA	1 (6.3)	0.314	0.575	44.862	NA	0 (0.0)	0.579	0.447	34.850	NA
B39(16)	39:06	0 (0.0)	0 (0.0)	NA	NA	NA	NA	0 (0.0)	NA	NA	NA	NA	0 (0.0)	NA	NA	NA	NA
B60(40)	40:01	13 (6.5)	2 (6.3)	0.003	0.957	74.679	NA	1 (6.3)	0.002	0.969	75.568	NA	1 (6.3)	0.002	0.969	75.568	NA
B61(40)	40:02	14 (7.0)	4 (12.5)	1.166	0.280	21.857	NA	3 (18.8)	2.821	0.093	7.258	NA	1 (6.3)	0.013	0.910	70.948	NA
B61(40)	40:03	4 (2.0)	1 (3.1)	0.166	0.684	53.358	NA	1 (6.3)	1.183	0.277	21.580	NA	0 (0.0)	0.326	0.568	44.304	NA
B61(40)	40:04	0 (0.0)	0 (0.0)	NA	NA	NA	NA	0 (0.0)	NA	NA	NA	NA	0 (0.0)	NA	NA	NA	NA
B61(40)	40:06	15 (7.5)	3 (9.4)	0.136	0.713	55.597	NA	2 (12.5)	0.511	0.475	37.034	NA	1 (6.3)	0.034	0.854	66.631	NA
B41	41:01	0 (0.0)	0 (0.0)	NA	NA	NA	NA	0 (0.0)	NA	NA	NA	NA	0 (0.0)	NA	NA	NA	NA

B41	41:02	0	(0.0)	0	(0.0)	NA	NA	NA	NA	0	(0.0)	NA	NA	NA	NA	0	(0.0)	NA	NA	NA	NA
B41	41:03	0	(0.0)	0	(0.0)	NA	NA	NA	NA	0	(0.0)	NA	NA	NA	NA	0	(0.0)	NA	NA	NA	NA
B44(12)	44:02	7	(3.5)	1	(3.1)	0.012	0.914	71.295	NA	0	(0.0)	0.579	0.447	34.850	NA	1	(6.3)	0.314	0.575	44.862	NA
B44(12)	44:03	31	(15.5)	7	(21.9)	0.819	0.366	28.517	NA	3	(18.8)	0.118	0.731	57.036	NA	4	(25.0)	0.985	0.321	25.041	NA
B44(12)	44:05	0	(0.0)	0	(0.0)	NA	NA	NA	NA	0	(0.0)	NA	NA	NA	NA	0	(0.0)	NA	NA	NA	NA
B44(12)	44:06	0	(0.0)	0	(0.0)	NA	NA	NA	NA	0	(0.0)	NA	NA	NA	NA	0	(0.0)	NA	NA	NA	NA
B46	46:01	22	(11.0)	4	(12.5)	0.062	0.803	62.617	NA	1	(6.3)	0.351	0.553	43.163	NA	3	(18.8)	0.869	0.351	27.387	NA
B47	47:01	0	(0.0)	2	(6.3)	12.609	**0.000**	**0.030**	NA	1	(6.3)	12.558	**0.000**	**0.031**	NA	1	(6.3)	12.558	**0.000**	**0.031**	NA
B48	48:01	15	(7.5)	3	(9.4)	0.136	0.713	55.597	NA	1	(6.3)	0.034	0.854	66.631	NA	2	(12.5)	0.511	0.475	37.034	NA
B48	48:03	0	(0.0)	0	(0.0)	NA	NA	NA	NA	0	(0.0)	NA	NA	NA	NA	0	(0.0)	NA	NA	NA	NA
B49(21)	49:01	0	(0.0)	0	(0.0)	NA	NA	NA	NA	0	(0.0)	NA	NA	NA	NA	0	(0.0)	NA	NA	NA	NA
B50(21)	50:01	0	(0.0)	0	(0.0)	NA	NA	NA	NA	0	(0.0)	NA	NA	NA	NA	0	(0.0)	NA	NA	NA	NA
B51(5)	51:01	37	(18.5)	5	(15.6)	0.154	0.695	54.205	NA	2	(12.5)	0.360	0.548	42.763	NA	3	(18.8)	0.001	0.980	76.458	NA
B5102	51:02	4	(2.0)	0	(0.0)	0.651	0.420	32.735	NA	0	(0.0)	0.326	0.568	44.304	NA	0	(0.0)	0.326	0.568	44.304	NA
B51(5)	51:07	0	(0.0)	0	(0.0)	NA	NA	NA	NA	0	(0.0)	NA	NA	NA	NA	0	(0.0)	NA	NA	NA	NA
B51(5)	51:08	0	(0.0)	0	(0.0)	NA	NA	NA	NA	0	(0.0)	NA	NA	NA	NA	0	(0.0)	NA	NA	NA	NA
B52(5)	52:01	9	(4.5)	2	(6.3)	0.187	0.665	51.900	NA	2	(12.5)	1.962	0.161	12.584	NA	0	(0.0)	0.751	0.386	30.113	NA
B52	52:05	0	(0.0)	0	(0.0)	NA	NA	NA	NA	0	(0.0)	NA	NA	NA	NA	0	(0.0)	NA	NA	NA	NA
B52(5)	52:06	0	(0.0)	0	(0.0)	NA	NA	NA	NA	0	(0.0)	NA	NA	NA	NA	0	(0.0)	NA	NA	NA	NA
B54(22)	54:01	26	(13.0)	2	(6.3)	1.184	0.276	21.565	NA	1	(6.3)	0.617	0.432	33.705	NA	1	(6.3)	0.617	0.432	33.705	NA
B55(22)	55:01	0	(0.0)	0	(0.0)	NA	NA	NA	NA	0	(0.0)	NA	NA	NA	NA	0	(0.0)	NA	NA	NA	NA
B55(22)	55:02	5	(2.5)	1	(3.1)	0.043	0.836	65.220	NA	0	(0.0)	0.409	0.522	40.734	NA	1	(6.3)	0.771	0.380	29.622	NA
B55(22)	55:04	0	(0.0)	1	(3.1)	6.277	**0.012**	0.954	NA	0	(0.0)	NA	NA	NA	NA	1	(6.3)	12.558	**0.000**	**0.031**	NA
B54(22)	55:07	1	(0.5)	0	(0.0)	0.161	0.689	53.705	NA	0	(0.0)	0.080	0.777	60.590	NA	0	(0.0)	0.080	0.777	60.590	NA
B56(22)	56:01	1	(0.5)	0	(0.0)	0.161	0.689	53.705	NA	0	(0.0)	0.080	0.777	60.590	NA	0	(0.0)	0.080	0.777	60.590	NA
B56(22)	56:02	0	(0.0)	0	(0.0)	NA	NA	NA	NA	0	(0.0)	NA	NA	NA	NA	0	(0.0)	NA	NA	NA	NA
B57(17)	57:01	2	(1.0)	0	(0.0)	0.323	0.570	44.455	NA	0	(0.0)	0.161	0.688	53.647	NA	0	(0.0)	0.161	0.688	53.647	NA
B57(17)	57:08	0	(0.0)	0	(0.0)	NA	NA	NA	NA	0	(0.0)	NA	NA	NA	NA	0	(0.0)	NA	NA	NA	NA
B58(17)	58:01	21	(10.5)	1	(3.1)	1.748	0.186	14.518	NA	1	(6.3)	0.293	0.589	45.912	NA	0	(0.0)	1.861	0.173	13.456	NA
B59	59:01	8	(4.0)	1	(3.1)	0.057	0.812	63.327	NA	0	(0.0)	0.665	0.415	32.365	NA	1	(6.3)	0.188	0.665	51.849	NA
B67	67:01	3	(1.5)	0		0.486	0.486	37.876	NA	0	(0.0)	0.243	0.622	48.498	NA	0		0.243	0.622	48.498	NA
B73	73:01	0	(0.0)	0	(0.0)	NA	NA	NA	NA	0	(0.0)	NA	NA	NA	NA	0	(0.0)	NA	NA	NA	NA
B81	81:01	1	(0.5)	0	(0.0)	0.161	0.689	53.705	NA	0	(0.0)	0.080	0.777	60.590	NA	0	(0.0)	0.080	0.777	60.590	NA

P_c, Bonferroni's correction; NA, not applicable

Table S3. Genetic influence of HLA-Bw4 in MERS patients

		Controls	MERS total					Mo/Mi cases					Severe cases				
Locus	Alleles	n = 200 (%)	n = 32 (%)	χ^2	p-value	P_c	OR	n = 16 (%)	χ^2	p-value	P_c	OR	n = 16 (%)	χ^2	p-value	P_c	OR
Bw4	13:01	9 (4.5)	1 (3.1)	0.126	0.722	24.553	NA	1 (6.3)	0.103	0.749	25.451	NA	0 (0.0)	0.751	0.386	13.126	NA
Bw4	15:13	0 (0.0)	0 (0.0)	NA	NA	NA	NA	0 (0.0)	NA	NA	NA	NA	0 (0.0)	NA	NA	NA	NA
Bw4	15:16	0 (0.0)	0 (0.0)	NA	NA	NA	NA	0 (0.0)	NA	NA	NA	NA	0 (0.0)	NA	NA	NA	NA
Bw4	15:17	0 (0.0)	0 (0.0)	NA	NA	NA	NA	0 (0.0)	NA	NA	NA	NA	0 (0.0)	NA	NA	NA	NA
Bw4	15:24	0 (0.0)	0 (0.0)	NA	NA	NA	NA	0 (0.0)	NA	NA	NA	NA	0 (0.0)	NA	NA	NA	NA
Bw4	24:03	0 (0.0)	0 (0.0)	NA	NA	NA	NA	0 (0.0)	NA	NA	NA	NA	0 (0.0)	NA	NA	NA	NA
Bw4	25:01	0 (0.0)	0 (0.0)	NA	NA	NA	NA	0 (0.0)	NA	NA	NA	NA	0 (0.0)	NA	NA	NA	NA
Bw4	27:09	0 (0.0)	0 (0.0)	NA	NA	NA	NA	0 (0.0)	NA	NA	NA	NA	0 (0.0)	NA	NA	NA	NA
Bw4	32:01	0 (0.0)	0 (0.0)	NA	NA	NA	NA	0 (0.0)	NA	NA	NA	NA	0 (0.0)	NA	NA	NA	NA
Bw4	38:02	7 (3.5)	1 (3.1)	0.012	0.914	31.077	NA	1 (6.3)	0.314	0.575	19.555	NA	0 (0.0)	0.579	0.447	15.191	NA
Bw4	44:03	31 (15.5)	7 (21.9)	0.819	0.366	12.431	NA	3 (18.8)	0.118	0.731	24.862	NA	4 (25.0)	0.985	0.321	10.915	NA
Bw4	47:01	0 (0.0)	2 (6.3)	12.609	0.257	8.722	NA	1 (6.3)	12.558	0.209	7.098	NA	1 (6.3)	12.558	0.209	7.098	NA
Bw4	52:01	9 (4.5)	2 (6.3)	0.187	0.665	22.623	NA	2 (12.5)	1.962	0.161	5.485	NA	0 (0.0)	0.751	0.386	13.126	NA
Bw4	53:01	0 (0.0)	0 (0.0)	NA	NA	NA	NA	0 (0.0)	NA	NA	NA	NA	0 (0.0)	NA	NA	NA	NA
Bw4	53:02	0 (0.0)	0 (0.0)	NA	NA	NA	NA	0 (0.0)	NA	NA	NA	NA	0 (0.0)	NA	NA	NA	NA
Bw4	57:03	0 (0.0)	0 (0.0)	NA	NA	NA	NA	0 (0.0)	NA	NA	NA	NA	0 (0.0)	NA	NA	NA	NA
Bw4	58:01	21 (10.5)	1 (3.1)	1.748	0.186	6.328	NA	1 (6.3)	0.293	0.589	20.013	NA	0 (0.0)	1.861	0.173	5.866	NA
Bw4	27:04	1 (0.5)	1 (3.1)	2.224	0.136	4.619	NA	1 (6.3)	5.339	**0.021**	0.709	NA	0 (0.0)	0.080	0.777	26.411	NA
Bw4	27:06	0 (0.0)	0 (0.0)	NA	NA	NA	NA	0 (0.0)	NA	NA	NA	NA	0 (0.0)	NA	NA	NA	NA
Bw4	51:02	4 (2.0)	0 (0.0)	0.651	0.420	14.269	NA	0 (0.0)	0.326	0.568	19.312	NA	0 (0.0)	0.326	0.568	19.312	NA
Bw4-80I	23:01	0 (0.0)	0 (0.0)	NA	NA	NA	NA	0 (0.0)	NA	NA	NA	NA	0 (0.0)	NA	NA	NA	NA
Bw4-80I	24:02	75 (37.5)	14 (43.8)	0.456	0.500	16.987	NA	7 (43.8)	0.246	0.620	21.084	NA	7 (43.8)	0.246	0.620	21.084	NA
Bw4-80I	24:07	0 (0.0)	0 (0.0)	NA	NA	NA	NA	0 (0.0)	NA	NA	NA	NA	0 (0.0)	NA	NA	NA	NA
Bw4-80I	25:01	0 (0.0)	0 (0.0)	NA	NA	NA	NA	0 (0.0)	NA	NA	NA	NA	0 (0.0)	NA	NA	NA	NA
Bw4-80I	32:01	1 (0.5)	0 (0.0)	0.161	0.689	23.410	NA	0 (0.0)	0.080	0.777	26.411	NA	0 (0.0)	0.080	0.777	26.411	NA
Bw4-80I	27:02	0 (0.0)	0 (0.0)	NA	NA	NA	NA	0 (0.0)	NA	NA	NA	NA	0 (0.0)	NA	NA	NA	NA
Bw4-80I	38:01	0 (0.0)	0 (0.0)	NA	NA	NA	NA	0 (0.0)	NA	NA	NA	NA	0 (0.0)	NA	NA	NA	NA
Bw4-80I	49:01	0 (0.0)	0 (0.0)	NA	NA	NA	NA	0 (0.0)	NA	NA	NA	NA	0 (0.0)	NA	NA	NA	NA
Bw4-80I	51:01	37 (18.5)	5 (15.6)	0.154	0.695	23.628	NA	2 (12.5)	0.360	0.548	18.640	NA	3 (18.8)	0.001	0.980	33.328	NA
Bw4-80I	57:01	2 (1.0)	0 (0.0)	0.323	0.570	19.378	NA	0 (0.0)	0.161	0.688	23.385	NA	0 (0.0)	0.161	0.688	23.385	NA
Bw4-80T	13:02	14 (7.0)	2 (6.3)	0.024	0.876	29.800	NA	0 (0.0)	1.198	0.274	9.309	NA	2 (12.5)	0.653	0.419	14.243	NA
Bw4-80T	27:05	15 (7.5)	0 (0.0)	2.566	0.109	3.712	NA	0 (0.0)	1.290	0.256	8.708	NA	0 (0.0)	1.290	0.256	8.708	NA
Bw4-80T	37:01	4 (2.0)	2 (6.3)	1.978	0.160	5.427	NA	1 (6.3)	1.183	0.277	9.407	NA	1 (6.3)	1.183	0.277	9.407	NA
Bw4-80T	44:02	7 (3.5)	1 (3.1)	0.012	0.914	31.077	NA	0 (0.0)	0.579	0.447	15.191	NA	1 (6.3)	0.314	0.575	19.555	NA

P_c, Bonferroni's correction; NA, not applicable

Bw4-80I or -80T, KIR3DL1 ligand HLA-B subtypes

Table S4. Genetic influence of HLA-Bw6 in MERS patients

		Controls	MERS total					Mo/Mi cases					Severe cases				
Locus	Alleles	n = 200 (%)	n = 32 (%)	χ^2	p-value	P_c	OR	n = 16 (%)	χ^2	p-value	P_c	OR	n = 16 (%)	χ^2	p-value	P_c	OR
Bw6	07:01	0 (0.0)	0 (0.0)	NA	NA	NA	NA	0 (0.0)	NA	NA	NA	NA	0 (0.0)	NA	NA	NA	NA
Bw6	07:02	18 (9.0)	2 (6.3)	0.265	0.607	21.846	NA	1 (6.3)	0.140	0.709	25.511	NA	1 (6.3)	0.140	0.709	25.511	NA
Bw6	07:10	0 (0.0)	0 (0.0)	NA	NA	NA	NA	0 (0.0)	NA	NA	NA	NA	0 (0.0)	NA	NA	NA	NA
Bw6	08:01	2 (1.0)	0 (0.0)	0.323	0.570	20.518	NA	0 (0.0)	0.161	0.688	24.760	NA	0 (0.0)	0.161	0.688	24.760	NA
Bw6	14:01	8 (4.0)	0 (0.0)	1.326	0.250	8.984	NA	0 (0.0)	0.665	0.415	14.938	NA	0 (0.0)	0.665	0.415	14.938	NA
Bw6	14:02	0 (0.0)	0 (0.0)	NA	NA	NA	NA	0 (0.0)	NA	NA	NA	NA	0 (0.0)	NA	NA	NA	NA
Bw6	15:01	34 (17.0)	4 (12.5)	0.408	0.523	18.830	NA	3 (18.8)	0.032	0.858	30.892	NA	1 (6.3)	1.261	0.261	9.413	NA
Bw6	15:02	1 (0.5)	0 (0.0)	0.161	0.689	24.787	NA	0 (0.0)	0.080	0.777	27.965	NA	0 (0.0)	0.080	0.777	27.965	NA
Bw6	15:03	0 (0.0)	0 (0.0)	NA	NA	NA	NA	0 (0.0)	NA	NA	NA	NA	0 (0.0)	NA	NA	NA	NA
Bw6	15:04	0 (0.0)	0 (0.0)	NA	NA	NA	NA	0 (0.0)	NA	NA	NA	NA	0 (0.0)	NA	NA	NA	NA
Bw6	15:07	3 (1.5)	1 (3.1)	0.430	0.512	18.433	NA	0 (0.0)	0.243	0.622	22.384	NA	1 (6.3)	1.839	0.175	6.302	NA
Bw6	15:09	0 (0.0)	0 (0.0)	NA	NA	NA	NA	0 (0.0)	NA	NA	NA	NA	0 (0.0)	NA	NA	NA	NA
Bw6	15:10	0 (0.0)	0 (0.0)	NA	NA	NA	NA	0 (0.0)	NA	NA	NA	NA	0 (0.0)	NA	NA	NA	NA
Bw6	15:15	0 (0.0)	0 (0.0)	NA	NA	NA	NA	0 (0.0)	NA	NA	NA	NA	0 (0.0)	NA	NA	NA	NA
Bw6	15:18	8 (4.0)	0 (0.0)	1.326	0.250	8.984	NA	0 (0.0)	0.665	0.415	14.938	NA	0 (0.0)	0.665	0.415	14.938	NA
Bw6	15:22	0 (0.0)	0 (0.0)	NA	NA	NA	NA	0 (0.0)	NA	NA	NA	NA	0 (0.0)	NA	NA	NA	NA
Bw6	15:37	0 (0.0)	0 (0.0)	NA	NA	NA	NA	0 (0.0)	NA	NA	NA	NA	0 (0.0)	NA	NA	NA	NA
Bw6	15:38	0 (0.0)	0 (0.0)	NA	NA	NA	NA	0 (0.0)	NA	NA	NA	NA	0 (0.0)	NA	NA	NA	NA
Bw6	15:45	0 (0.0)	0 (0.0)	NA	NA	NA	NA	0 (0.0)	NA	NA	NA	NA	0 (0.0)	NA	NA	NA	NA
Bw6	15:48	0 (0.0)	0 (0.0)	NA	NA	NA	NA	0 (0.0)	NA	NA	NA	NA	0 (0.0)	NA	NA	NA	NA
Bw6	18:03	0 (0.0)	0 (0.0)	NA	NA	NA	NA	0 (0.0)	NA	NA	NA	NA	0 (0.0)	NA	NA	NA	NA
Bw6	27:05	15 (7.5)	0 (0.0)	2.566	0.109	3.931	NA	0 (0.0)	1.290	0.256	9.221	NA	0 (0.0)	1.290	0.256	9.221	NA
Bw6	35:01	18 (9.0)	3 (9.4)	0.005	0.945	34.030	NA	2 (12.5)	0.216	0.642	23.116	NA	1 (6.3)	0.140	0.709	25.511	NA
Bw6	35:08	0 (0.0)	0 (0.0)	NA	NA	NA	NA	0 (0.0)	NA	NA	NA	NA	0 (0.0)	NA	NA	NA	NA
Bw6	39:10	0 (0.0)	0 (0.0)	NA	NA	NA	NA	0 (0.0)	NA	NA	NA	NA	0 (0.0)	NA	NA	NA	NA
Bw6	40:01	13 (6.5)	2 (6.3)	0.003	0.957	34.467	NA	1 (6.3)	0.002	0.969	34.878	NA	1 (6.3)	0.002	0.969	34.878	NA
Bw6	41:01	0 (0.0)	0 (0.0)	NA	NA	NA	NA	0 (0.0)	NA	NA	NA	NA	0 (0.0)	NA	NA	NA	NA
Bw6	41:03	0 (0.0)	0 (0.0)	NA	NA	NA	NA	0 (0.0)	NA	NA	NA	NA	0 (0.0)	NA	NA	NA	NA
Bw6	45:01	0 (0.0)	0 (0.0)	NA	NA	NA	NA	0 (0.0)	NA	NA	NA	NA	0 (0.0)	NA	NA	NA	NA
Bw6	46:01	22 (11.0)	4 (12.5)	0.062	0.803	28.900	NA	1 (6.3)	0.351	0.553	19.921	NA	3 (18.8)	0.869	0.351	12.640	NA
Bw6	51:01	37 (18.5)	5 (15.6)	0.154	0.695	25.018	NA	2 (12.5)	0.360	0.548	19.737	NA	3 (18.8)	0.001	0.980	35.289	NA
Bw6	54:01	26 (13.0)	2 (6.3)	1.184	0.276	9.953	NA	1 (6.3)	0.617	0.432	15.556	NA	1 (6.3)	0.617	0.432	15.556	NA
Bw6	73:01	0 (0.0)	0 (0.0)	NA	NA	NA	NA	0 (0.0)	NA	NA	NA	NA	0 (0.0)	NA	NA	NA	NA
Bw6	78:01	0 (0.0)	0 (0.0)	NA	NA	NA	NA	0 (0.0)	NA	NA	NA	NA	0 (0.0)	NA	NA	NA	NA
Bw6	81:01	1 (0.5)	0 (0.0)	0.161	0.689	24.787	NA	0 (0.0)	0.080	0.777	27.965	NA	0 (0.0)	0.080	0.777	27.965	NA
Bw6	82:01	0 (0.0)	0 (0.0)	NA	NA	NA	NA	0 (0.0)	NA	NA	NA	NA	0 (0.0)	NA	NA	NA	NA

P_c, Bonferroni's correction; NA, not applicable

Table S5. Genetic influence of HLA-C in MERS patients

HLA-C1/C2 group	HLA-C alleles	Controls n = 200 (%)	MERS total n = 32 (%)	χ^2	p-value	P_c	OR	Mo/Mi cases n = 16 (%)	χ^2	p-value	P_c	OR	Severe cases n = 16 (%)	χ^2	p-value	P_c	OR
C1	C*01:02	72 (36.0)	7 (21.9)	2.451	0.117	2.467	NA	1 (6.3)	5.860	**0.009**	0.193	**0.1**	6 (37.5)	0.014	0.904	18.991	NA
C1	C*01:03	3 (1.5)	1 (3.1)	0.430	0.512	10.753	NA	0 (0.0)	0.243	0.622	13.057	NA	1 (6.3)	1.839	0.175	3.676	NA
C1	C*03:02	21 (10.5)	1 (3.1)	1.748	0.186	3.909	NA	1 (6.3)	0.293	0.589	12.361	NA	0 (0.0)	1.861	0.173	3.623	NA
C1	C*03:03	36 (18.0)	7 (21.9)	0.274	0.600	12.609	NA	3 (18.8)	0.006	0.940	19.744	NA	4 (25.0)	0.481	0.488	10.247	NA
C1	C*03:04	34 (17.0)	6 (18.8)	0.059	0.808	16.963	NA	5 (31.3)	2.033	0.154	3.232	NA	1 (6.3)	1.261	0.261	5.491	NA
C1	C*07:02	36 (18.0)	3 (9.4)	1.467	0.226	4.741	NA	2 (12.5)	0.309	0.578	12.143	NA	1 (6.3)	1.441	0.230	4.830	NA
C1	C*07:04	8 (4.0)	0 (0.0)	1.326	0.250	5.241	NA	0 (0.0)	0.665	0.415	8.714	NA	0 (0.0)	0.665	0.415	8.714	NA
C1	C*07:06	8 (4.0)	4 (12.5)	4.064	0.052	0.920	NA	1 (6.3)	0.188	0.512	10.758	NA	3 (18.8)	6.669	0.512	10.758	NA
C1	C*08:01	30 (15.0)	7 (21.9)	0.973	0.324	6.804	NA	4 (25.0)	1.117	0.291	6.102	NA	3 (18.8)	0.161	0.688	14.454	NA
C1	C*08:02	8 (4.0)	0 (0.0)	1.326	0.250	5.241	NA	0 (0.0)	0.665	0.415	8.714	NA	0 (0.0)	0.665	0.415	8.714	NA
C1	C*08:03	3 (1.5)	0 (0.0)	0.486	0.486	10.197	NA	0 (0.0)	0.243	0.622	13.057	NA	0 (0.0)	0.243	0.622	13.057	NA
C1	C*12:02	10 (5.0)	3 (9.4)	0.998	0.318	6.672	NA	3 (18.8)	4.952	0.051	0.876	NA	0 (0.0)	0.839	0.360	7.554	NA
C1	C*12:03	3 (1.5)	0 (0.0)	0.486	0.486	10.197	NA	0 (0.0)	0.243	0.622	13.057	NA	0 (0.0)	0.243	0.622	13.057	NA
C1	C*14:02	28 (14.0)	5 (15.6)	0.060	0.807	16.946	NA	2 (12.5)	0.028	0.867	18.216	NA	3 (18.8)	0.272	0.602	12.643	NA
C1	C*14:03	24 (12.0)	3 (9.4)	0.185	0.667	14.012	NA	2 (12.5)	0.003	0.953	20.010	NA	1 (6.3)	0.479	0.489	10.270	NA
C2	C*02:02	4 (2.0)	0 (0.0)	0.651	0.420	8.813	NA	0 (0.0)	0.326	0.568	11.928	NA	0 (0.0)	0.326	0.568	11.928	NA
C2	C*04:01	22 (11.0)	3 (9.4)	0.076	0.783	16.446	NA	3 (18.8)	0.869	0.351	7.373	NA	0 (0.0)	1.960	0.162	3.393	NA
C2	C*05:01	7 (3.5)	1 (3.1)	0.012	0.914	19.195	NA	0 (0.0)	0.579	0.447	9.383	NA	1 (6.3)	0.314	0.575	12.078	NA
C2	C*06:02	19 (9.5)	6 (18.8)	2.455	0.117	2.460	NA	2 (12.5)	0.152	0.697	14.631	NA	4 (25.0)	3.741	0.053	1.115	NA
C2	C*15:02	13 (6.5)	1 (3.1)	0.554	0.457	9.589	NA	0 (0.0)	1.107	0.293	6.149	NA	1 (6.3)	0.002	0.969	20.345	NA
C2	C*15:05	2 (1.0)	1 (3.1)	0.976	0.323	6.787	NA	1 (6.3)	2.981	0.084	1.769	NA	0 (0.0)	0.161	0.688	14.443	NA
C1C1		139 (69.5)	20 (62.5)	0.627	0.429	1.286	NA	10 (62.5)	0.339	0.560	1.681	NA	10 (62.5)	0.339	0.560	1.681	NA
C1C2		55 (27.5)	12 (37.5)	1.343	0.246	0.739	NA	8 (50.0)	3.630	0.057	0.170	NA	6 (37.5)	0.731	0.393	1.178	NA
C2C2		6 (3.0)	0 (0.0)	0.985	0.321	0.963	NA	0 (0.0)	0.494	0.482	1.447	NA	0 (0.0)	0.494	0.482	1.447	NA

P_c, Bonferroni's correction; NA, not applicable; ¥, Fisher exact test

C1 group: Ser77Asn80 allele among HLA-C alleles.

C2 group: Asn77Lys80 allele among HLA-C alleles.

Table S6. Genetic influence of KIR haplotypes in MERS patients

Haplotype group	Genotype ID	Controls	MERS total				Moderate/Mild cases				Severe cases			
		n = 200 (%)	n = 32 (%)	χ^2	p-value	OR	n = 16 (%)	χ^2	p-value	OR	n = 16 (%)	χ^2	p-value	OR
AA	1	111 (55.5)	11 (34.4)	4.938	**0.026**	**0.4**	6 (37.5)	1.933	0.164	NA	5 (31.3)	3.504	0.061	NA
AA	180	1 (0.5)	0 (0.0)	0.161	0.689	NA	0 (0.0)	0.080	0.777	NA	0 (0.0)	0.080	0.777	NA
Bx	2	27 (13.5)	5 (15.6)	0.105	0.746	NA	4 (25.0)	1.594	0.207	NA	1 (6.3)	0.690	0.406	NA
Bx	8	19 (9.5)	6 (18.8)	2.455	0.117	NA	3 (18.8)	1.386	0.239	NA	3 (18.8)	1.386	0.239	NA
Bx	4	9 (4.5)	3 (9.4)	1.337	0.248	NA	0 (0.0)	0.751	0.386	NA	3 (18.8)	5.734	**0.042**	**4.9**
Bx	3	3 (1.5)	2 (6.3)	2.952	0.086	NA	1 (6.3)	1.839	0.175	NA	1 (6.3)	1.839	0.175	NA
Bx	70	3 (1.5)	1 (3.1)	0.430	0.512	NA	1 (6.3)	1.839	0.175	NA	0 (0.0)	-	-	-
Bx	154	1 (0.5)	1 (3.1)	2.224	0.136	NA	0 (0.0)	0.080	0.777	NA	1 (6.3)	5.339	0.138	NA
Bx	7	0 (0.0)	1 (3.1)	6.277	0.052	NA	0 (0.0)	-	-	-	1 (6.3)	12.558	**0.018**	**25.1**
Bx	36	0 (0.0)	1 (3.1)	6.277	0.052	NA	0 (0.0)	-	-	-	1 (6.3)	12.558	**0.018**	**25.1**
Bx	72	0 (0.0)	1 (3.1)	6.277	0.052	NA	1 (6.3)	12.558	**0.018**	**25.1**	0 (0.0)	-	-	-
Bx	75	4 (2.0)	0 (0.0)	0.651	0.420	NA	0 (0.0)	0.326	0.568	NA	0 (0.0)	0.326	0.568	NA
Bx	10	4 (2.0)	0 (0.0)	0.651	0.420	NA	0 (0.0)	0.326	0.568	NA	0 (0.0)	0.326	0.568	NA
Bx	69	4 (2.0)	0 (0.0)	0.651	0.420	NA	0 (0.0)	0.326	0.568	NA	0 (0.0)	0.326	0.568	NA
Bx	13	2 (1.0)	0 (0.0)	0.323	0.570	NA	0 (0.0)	0.161	0.688	NA	0 (0.0)	0.161	0.688	NA
Bx	68	2 (1.0)	0 (0.0)	0.323	0.570	NA	0 (0.0)	0.161	0.688	NA	0 (0.0)	0.161	0.688	NA
Bx	159	2 (1.0)	0 (0.0)	0.323	0.570	NA	0 (0.0)	0.161	0.688	NA	0 (0.0)	0.161	0.688	NA
Bx	6	2 (1.0)	0 (0.0)	0.323	0.570	NA	0 (0.0)	0.161	0.688	NA	0 (0.0)	0.161	0.688	NA
Bx	56	1 (0.5)	0 (0.0)	0.161	0.689	NA	0 (0.0)	0.080	0.777	NA	0 (0.0)	0.080	0.777	NA
Bx	331	1 (0.5)	0 (0.0)	0.161	0.689	NA	0 (0.0)	0.080	0.777	NA	0 (0.0)	0.080	0.777	NA
Bx	11	1 (0.5)	0 (0.0)	0.161	0.689	NA	0 (0.0)	0.080	0.777	NA	0 (0.0)	0.080	0.777	NA
Bx	5	1 (0.5)	0 (0.0)	0.161	0.689	NA	0 (0.0)	0.080	0.777	NA	0 (0.0)	0.080	0.777	NA
Bx	16	1 (0.5)	0 (0.0)	0.161	0.689	NA	0 (0.0)	0.080	0.777	NA	0 (0.0)	0.080	0.777	NA
Bx	166	1 (0.5)	0 (0.0)	0.161	0.689	NA	0 (0.0)	0.080	0.777	NA	0 (0.0)	0.080	0.777	NA

NA, not applicable
